# Influence of Obesity in Children with Supracondylar Humeral Fractures Requiring Surgical Treatment at a Tertiary Pediatric Trauma Center

**DOI:** 10.3390/healthcare11121783

**Published:** 2023-06-16

**Authors:** Marko Bašković, Lucija Vucković, Marta Borić Krakar, Arnes Rešić, Nikolina Benco Kordić, Antun Kljenak

**Affiliations:** 1 Department of Pediatric Surgery, Children’s Hospital Zagreb, Ulica Vjekoslava Klaića 16, 10 000 Zagreb, Croatia; 2 Scientific Centre of Excellence for Reproductive and Regenerative Medicine, School of Medicine, University of Zagreb, Šalata 3, 10 000 Zagreb, Croatia; 3 School of Medicine, University of Zagreb, Šalata 3, 10 000 Zagreb, Croatia; 4 University of Applied Health Sciences, Mlinarska Cesta 38, 10 000 Zagreb, Croatia; 5 Department of Pediatrics, Children’s Hospital Zagreb, Ulica Vjekoslava Klaića 16, 10 000 Zagreb, Croatia; 6 University Department of Health Studies, University of Split, Ruđera Boškovića 35, 21 000 Split, Croatia

**Keywords:** obesity, overweight, children, supracondylar fracture, humerus, trauma

## Abstract

Background: Almost everywhere in the world, childhood obesity is becoming a serious public health problem with negative effects on both children’s health and society as a whole. The main objective of this study was to determine whether obesity has an effect on the severity of supracondylar humerus fracture in children, regardless of whether it is a low- or high-energy trauma. Methods: The electronic records of patients treated for the supracondylar fracture of the humerus in the ten-year period from 1 January 2013 to 1 January 2023 were reviewed retrospectively. Results: In the observed period, 618 children, including 365 (59.06%) boys and 253 (40.94%) girls, were hospitalized and treated surgically with the diagnosis of supracondylar fracture. The distributions according to the observed parameters were as follows: age (months) = 88.18 ± 32.64; height (cm) = 123.42 ± 16.83; weight (kg) = 27.18 ± 11.32; body mass index = 17.18 ± 3.06; body mass index-for-age percentile = 57.34 ± 32.11. Overall, 141 (22.82%) fractures were classified as Gartland II, while 477 (77.18%) were classified as Gartland III. A total of 66 (10.68%) fractures were flexion type, while 552 (89.32%) were extension type. The left elbow was affected in 401 (64.89%) children, while the right was affected in 217 (35.11%) children. The main mechanism of injury was a fall at ground level (33.33%). In relation to gender, a statistically significant difference was recorded in body mass index and percentile (*p* < 0.05). According to Gartland, the proportion of children below and above the 85th percentile in relation to the type of injury was statistically significant (*p* < 0.05). It was determined that the energy level does not significantly influence the injury’s severity: *p*(GII) = 0.225; *p*(GIII) = 0.180. Conclusions: In our study, we found that the proportion of overweight and obese children requiring surgical treatment was higher in Gartland type III injury, so there is no doubt that as a society we must prevent further increases in the prevalence of childhood obesity for this reason as well.

## 1. Introduction

Obesity is one of today’s biggest public health problems in both highly developed and underdeveloped and developing countries. Obesity in childhood is a growing problem for the health and well-being of the child [1]. The etiology of obesity is complex, and there is growing evidence of influences during early life and even the prenatal period, such as epigenetic factors, prenatal environment, maternal obesity, etc. [2,3,4]. The prevalence of overweight and obesity in children aged 5 to 19 years increased dramatically from 4% in 1975 to just over 18% in 2016. Currently, in 2022, 340 million adolescents and 39 million children under the age of 5 are obese. By 2025, the WHO predicts that 167 million adults and children will be less healthy due to being overweight or obese [5]. In the Republic of Croatia, the share of overweight and obese children increased from 20.8% in 2003 to 34.9% in 2015 [6]. Obesity in children has a number of serious health and social consequences and is also strongly associated with risk factors for orthopedic problems. Key areas for intervention are schools, homes, neighborhoods, primary health care centers, communities, media, and the food industry [7].

Supracondylar fractures of the humerus, with a proportion of 55–80% of all elbow fractures, are the most common fractures in the elbow area in children. This type of fracture accounts for up to 2/3 of all hospitalizations due to fractures in the elbow area. They most often occur as a result of falling from a height or during sports activities. The frequency is estimated at 177.3 cases per 100,000 children [8,9,10].

The main aim of our research was to determine whether the proportion of overweight and obese children, hospitalized for the surgical treatment of supracondylar humerus fracture, was higher in the more severe form of supracondylar fracture with complete displacement.

## 2. Materials and Methods

### 2.1. Patients

For the purpose of the research, two independent researchers retrospectively analyzed patient electronic records in the hospital information system (BIS^®^, IN2 Group, ver. 209.0.000) (the inter-rater reliability, evaluated using intraclass correlation coefficients, was 0.936; 95% CI: 0.914–0.952). In the case of vagueness, a third researcher was also involved. The search included all patients treated at Children’s Hospital Zagreb with a diagnosis of S42.4 (fracture of the lower end of humerus—according to the International Classification of Diseases 10th Revision) in the period from 1 January 2013 to 1 January 2023. The inclusion criteria were all patients aged 0 to 18 years, including boys and girls. The surgery was performed immediately after the clinical and radiological examination (anteroposterior and lateral radiographs). Prior to fixation, the surgeon assessed the position of the fragments under fluoroscopy. After closed or open reduction, the surgeon introduced two (one laterally and one medially) or three (three laterally or two laterally and one medially) Kirshner wires, depending on the stability. Once the fracture was fixed, the surgeon manipulated the elbow and checked for stability and the possibility of flexion and extension. The wires were cut, bent, and then buried under the skin. After surgery and three weeks of immobilization, the wires were usually removed. In a ten-year period, all interventions were performed by 11 pediatric surgeons.

### 2.2. Outcome Measures

The primary outcome of the study was to determine the cumulative number of all supracondylar fractures surgically treated in our institution and their distribution by age, sex, height, weight, body mass index (weight (kg)/height (m)^2^—according to BMI for child and teen, Centers for Disease Control and Prevention, Atlanta, GA, USA), body mass index-for-age percentile (which shows a child’s weight compared to that of other children of the same age and sex—according to BMI percentile for child and teen, Centers for Disease Control and Prevention, Atlanta, GA, USA), Gartland type (type 1—undisplaced fracture; type 2—angulated fracture with intact posterior cortex; type 3—displaced distal fragment with no cortical contact), flexion–extension type, elbow side, and mechanism of injury.

The secondary outcome was to determine whether there was a statistically significant difference in relation to the observed parameters depending on gender.

The tertiary outcome was to determine the proportion of children, depending on their percentile (<5th, 5th–85th, 85th–95th, and >95th), with Gartland type II and Gartland type III injuries and whether the level of energy affects the type of injuries (in low-energy trauma, according to the definition of the World Health Organization, we included fall causes equivalent to a fall from a standing height or less). We were also interested in whether the proportion of complications is higher in obese children with a more severe type of injury and whether overweight/obesity is associated with a lower injury (below the isthmus of the distal humerus) in Gartland type III injury.

The study’s main outcome was to determine whether the proportion of overweight and obese children (>85th) was higher in Gartland type III injury.

### 2.3. Statistical Analysis

The obtained data were analyzed using the Microsoft Excel^®^ software program (XLSTAT^®^) for Windows, version 2020.5.1 (Microsoft Corporation, Redmond, WA, USA). Variables of interest were assessed for normality using the Shapiro–Wilk test. Categorical variables were expressed in absolute numbers and percentages. Fisher’s exact test was used to assess differences in the distribution of categorical data. Continuous variables were expressed as mean with standard deviation (SD) and median (Mdn) with interquartile range (IQR) and were analyzed using Student’s *t*-test or Mann–Whitney U test as appropriate. The proportions are shown in percentages, while the existence of a difference between the observed groups was tested with the chi-square test. A significance level of 0.05 was used.

## 3. Results

**Figure 1:**
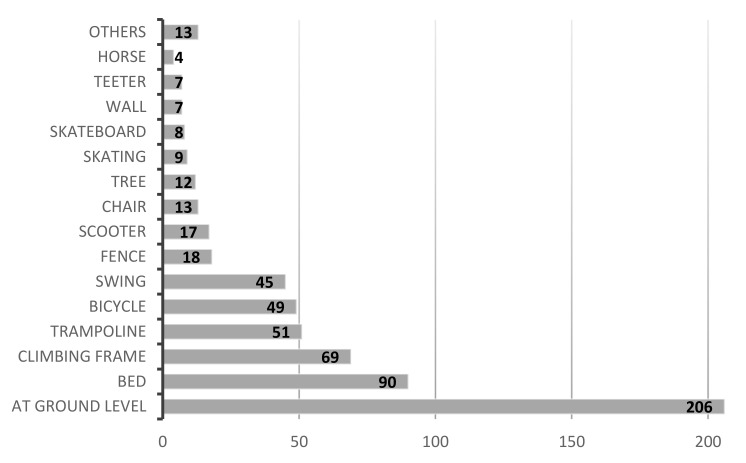
Mechanisms of injury.

In the observed period, 618 children, including 365 (59.06%) boys and 253 (40.94%) girls, were hospitalized. The distributions according to the observed parameters were as follows: age (months) = 88.18 ± 32.64; height (cm) = 123.42 ± 16.83; weight (kg) = 27.18 ± 11.32; body mass index = 17.18 ± 3.06; BMI-for-age percentile = 57.34 ± 32.11. Overall, 141 (22.82%) fractures were classified as Gartland II, while 477 (77.18%) were classified as Gartland III. A total of 66 (10.68%) fractures were flexion type, while 552 (89.32%) were extension type. The left elbow was affected in 401 (64.89%) children, while the right was affected in 217 (35.11%) children. The mechanisms of injury, i.e., how the child fell, are shown in Figure 1.

The observed parameters, and whether there is a statistically significant difference between them, depending on gender, are shown in Table 1.

In boys, there was a total of 83 (22.74%) type II fractures and 282 (77.26%) type III fractures. There were also 23 (6.30%) flexion and 342 (93.70%) extension types. The left elbow was affected in 211 (57.81%) boys, while the right was affected in 154 (42.19%) boys. In girls, there was a total of 58 (22.92%) type II fractures and 195 (77.08%) type III fractures. There were also 38 (15.02%) flexion and 215 (84.98%) extension types. The left elbow was affected in 185 (73.12%) girls, while the right was affected in 68 (26.88%) girls. According to the mechanisms of injury, fractures in boys were dominated by falls at ground level (*n* = 99), falls from a bicycle (*n* = 38), and falls from the bed and climber (*n* = 47). Fractures in girls were dominated by falls at ground level (*n* = 107), falls from bed (*n* = 43), and falls from a swing (*n* = 31). Depending on the percentile in which the children are distributed, Table 2 shows the distribution in relation to Gartland type II and type III injuries (*p* < 0.001).

According to the Gartland classification, the proportion of children below and above the 85th percentile in relation to the type of injury is shown in Table 3 (*p* < 0.001).

When Gartland type II and Gartland type III fractures were observed separately, depending on the percentiles (<, >85th), it was concluded that the level of energy does not have a statistically significant effect on the severity of the injury (*p* = 0.225 and *p* = 0.180, respectively) (Table 4).

The ORIF (open reduction and internal fixation) method was used in 9 patients (of whom 7 were overweight/obese), while a total of 13 patients (of whom 3 were overweight/obese) suffered an open fracture. A pin tract infection was observed in two patients (0.32%), both from the obesity group. A transient postoperative neurological deficit was verified in fourteen patients (2.27%), of whom eleven were in the group above the 85th percentile (all Gartland type III injury) (*p* = 0.115). Compartment syndrome was not recorded in any patient. Due to the loss of reduction in four patients, two below and two above the 85th percentile, revision surgery was required. According to Flynn’s criteria, functional outcomes were satisfactory in 98.71% patients (out of eight patients with worse functional outcomes, six were from the >85th percentile group; *p* = 0.302). The proportion of low type of injuries in Gartland type III fractures in children above the 85th percentile was not statistically significant (*p* = 0.227) (Table 5, Figure S1).

## 4. Discussion

In our study, we found a statistically significant difference in the proportion of Gartland type III injuries in overweight and obese children compared to children whose weight was below the 85th percentile. The impact of trauma energy was not statistically significant, regardless of the Gartland type of injury. The first studies on this topic clearly indicated that obese children have a higher incidence of extremity fractures and orthopedic surgical intervention [11]. A systematic review of published studies that evaluated the effects of obesity on children with traumatic injuries has pointed to the fact that obese children were 25% more likely to have extremity fractures than nonobese children [12]. In a Spanish cohort study of 466,997 children, cumulative fracture incidence was 9.2% for underweight patients, 10.06% for normal weight patients, 11.28% for overweight patients, and 13.05% for obese patients [13]. Compared with children of normal body weight, the body mass index of overweight and obesity was associated with an increased risk of upper and lower extremity fractures. According to a study by Nhan et al. [14], who observed all fractures of the upper extremities, children who were overweight or obese had a considerably higher percentage of complete fractures than children who were of normal weight (65% vs. 55%). In the mentioned study, in the American population, the proportion of overweight/obese children is far higher than in our study (42% vs. 26.5%), but, unlike ours, we note that, in the aforementioned study, all fractures of the upper extremities were observed. When we observe the mechanisms of injury, according to the study by Manning Ryan et al. [15], in the cohort of pediatric patients with a forearm fracture, a ground-level fall was a common mechanism of injury, accounting for 24% of all forearm fractures. In our study, ground-level falls accounted for 33.33%. Valerio et al. [16] noted that the greater prevalence of overweight/obesity was in girls and that low-energy trauma (such as falling to the ground from a standing height or from a bed, playing injuries, or sporting injuries) occurred in 71.1% of children, while moderate-energy trauma (such as falling down stairs, falling off a bicycle, or falling while moving on skateboards or rollerblades) occurred in 28.9% children. In articles on supracondylar fractures, we found a good correlation with our results. As we noted in our results, the study conducted by Nazareth et al. [17] showed that obese children, with completely displaced supracondylar humerus fractures, have an increased risk of Gartland type III/IV injury and that they have a significantly higher incidence of nerve palsy. A study by Li et al. [18] on a sample of 31,905 children showed that supracondylar humerus fractures sustained in obese children between the ages of 8 and 12 are over four times more likely to require the open reduction and internal fixation method (ORIF) than fractures sustained in patients of healthy weight. At the threshold of our findings, it was necessary to perform the ORIF method on seven obese patients compared to two of healthy weight. According to a study by DiBartola et al. [19], the average age of the operated children was 5.9 years, which is less than the average age in our study, which was 7.3 years. The average BMI of their patients was 17.2, which is almost identical to our reported average BMI. In our study, the percentage of obese children was slightly lower (17.32% vs. 21.5%), but the percentage of girls (45.8%) and boys (54.2%) was similar to our study (40.94% vs. 59.06%). Similarly to our results, the study also concluded that obesity was not associated with a significantly higher overall rate of complications, range of motion loss, or requirement of postoperative physical therapy. In the Chang et al. sample of 107 patients with Gartland type III fractures, 9.35% of children were underweight, 66.35% of children were of normal weight, 12.15% of children were overweight, and 12.15% of children were obese [20]. The mean age at the time of surgery was 6.2 years. As with our research, there were no significant differences in age, gender, and injured side, and obesity was associated with more postoperative varus deformations and pin-related complications after surgical fixation for type III supracondylar fractures. In the study conducted by Seeley et al., children with Gartland type III fractures had underweight at 11.58%, normal weight at 51.41%, overweight at 17.8%, and obese at 19.21% [21]. The proportion of obesity in type II fractures was 7.38%, while, in our study, it was 5.67%. In the mentioned study, it was concluded that obesity is associated with more complex supracondylar humeral fractures, preoperative and postoperative nerve palsies, and postoperative complications. Similarly to our study, 24.85% of children fell at ground level. Fornari et al. [22] observed that the lateral condyle fracture group had a higher mean BMI than the supracondylar group. It is interesting to note that, within the supracondylar group, there was no difference between the BMI or BMI-for-age percentiles of the children when analyzed by fracture subtype. There was also no difference in the percentage of obese and nonobese patients in each fracture subgroup. Twenty-three percent of the children included in this study were classified as obese. Similarly to the study by Kang et al. [23], we observed a similar proportion of high fracture types (86.1% vs. 87.2%) and low fracture types (13.9% vs. 12.8%) in Gartland type III supracondylar fractures. The other results of the observed parameters in the aforementioned study were similar to ours. We also come across similar results when we observe the impact of obesity on the forearm and lower extremities. Liu et al. [24] concluded that overweight/obesity, with an associated ulnar fracture and three-point index ≥0.40, also affects the outcome of conservative treatments of distal radius fractures, with a double probability of displacement. A study by Auer et al. [25] also emphasizes the hypothesis that obesity results in a higher rate of malreduction and subsequent manipulations with closed reduction and casting. Additionally, obese children had a significantly lower chance to have an initial perfect reduction in the emergency room. In our research, interestingly, we recorded only three open fractures in the group of obese children, compared to ten in healthy weight children. The answer probably lies in the fact of a thick soft tissue envelope. A study by Li et al. [26] also confirmed this fact on forearm fractures, for which they concluded that children with normal body weight were 4.1 times more likely to sustain an open fracture compared with obese children. The probable answer to the low pin tract infection rate is found in concealing the K-wires under the skin and removing them under general anesthesia under aseptic conditions. When tibial and femur fractures are observed in children, we also notice that the proportion of obese children is about 20%. Patients who were obese had double the risk of physeal fractures. Femur fractures carried a higher risk of physeal fracture than tibia fractures. Severity did not differ between groups [27]. Although the results of our study correlate with the results of other studies, we must also be aware of the limitations of our study. The study was retrospective and single-center. During the observed period, the surgery was performed by several different pediatric surgeons. It is also important to note that BMI is only an indicator of body fatness. Individuals with the same BMI may have different amounts of body fat.

## 5. Conclusions

Although most health problems are related to endocrine abnormalities and an increased risk of cardiovascular disease later in life, physicians should also be aware of orthopedic problems associated with childhood overweight, including an increased risk of fractures and a higher severity of fractures. Our study found that the proportion of overweight and obese children requiring surgical treatment was higher in Gartland type III injury. Additionally, a growing number of reports describe higher rates of complications associated with surgical and conservative treatment in overweight children. The health community as a whole may be further motivated to take specific measures to lower the prevalence of childhood obesity in light of these findings.

## Figures and Tables

**Table 1:** Observed parameters depending on gender.

	Boys (*n* = 365)	Girls (*n* = 253)	*p*-Value
Age (months)	87.60 (±34.96)	89.02 (±28.95)	0.362
Height (cm)	123.30 (±17.89)	123.60 (±15.16)	0.054
Weight (kg)	28.43 (±13.11)	25.39 (±7.72)	0.110
Body mass index *	17.81 (±3.36)	16.27 (±2.28)	<0.05
Percentile *	63.00 (±31.63)	49.17 (±31.02)	<0.05

* According to BMI and BMI percentile for child and teen, CDC, Atlanta, Georgia, USA.

**Table 2:** The proportion of children, depending on their percentile, according to Gartland type II and Gartland type III injuries.

	Gartland Type II (*n* = 141)	Gartland Type III (*n* = 477)	Total (*n* = 618)
**Underweight** (<5th percentile)	3 (2.13%)	5 (1.05%)	8 (1.29%)
**Healthy weight** (5th–85th percentile)	122 (86.53%)	324 (67.93%)	446 (72.17%)
**Overweight** (85th–95th percentile)	8 (5.67%)	49 (10.27%)	57 (9.22%)
**Obesity** (>95th percentile)	8 (5.67%)	99 (20.75%)	107 (17.32%)

**Table 3:** The proportion of children below and above the 85th percentile in relation to the type of injury according to Gartland classification.

	Gartland Type II (*n* = 141)	Gartland Type III (*n* = 477)
**Underweight/Healthy weight** (<85th percentile)	125 (88.65%)	329 (68.97%)
**Overweight/Obesity** (>85th percentile)	16 (11.35%)	148 (31.03%)

**Table 4:** The proportion of children below and above the 85th percentile in relation to trauma energy in Gartland type II and Gartland type III fractures.

Gartland Type II	Low-Energy (*n* = 73)	High-Energy (*n* = 68)
**Underweight/Healthy weight** (<85th percentile) (*n* = 125)	67 (91.78%)	58 (85.29%)
**Overweight/Obesity** (>85th percentile) (*n* = 16)	6 (8.22%)	10 (14.71%)
**Gartland type III**	**Low-energy (*n* = 236)**	**High-energy (*n* = 241)**
**Underweight/Healthy weight** (<85th percentile) (*n* = 329)	156 (66.10%)	173 (71.78%)
**Overweight/Obesity** (>85th percentile) (*n* = 148)	80 (33.90%)	68 (28.22%)

**Table 5:** The proportion of children below and above the 85th percentile in relation to the high and low types of injury in Gartland type III fracture.

	High Type	Low Type
**Underweight/Healthy weight** (<85th percentile) (*n* = 329)	291 (88.45%)	38 (11.55%)
**Overweight/Obesity** (>85th percentile) (*n* = 148)	125 (84.46%)	23 (15.54%)

## Data Availability

The data supporting this study’s findings are available upon request from the corresponding author.

## Supplementary Materials

The following supporting information can be downloaded at https://www.mdpi.com/article/10.3390/healthcare11121783/s1. Figure S1: Anteroposterior and lateral X-rays of supracondylar fractures.
