# Supplementary material for: Influence of Obesity in Children with Supracondylar Humeral Fractures Requiring Surgical Treatment at a Tertiary Pediatric Trauma Center
Source: Healthcare (Basel). 2023 Jun 16;11(12):1783. doi: 10.3390/healthcare11121783 (PMC10298723; doi:10.3390/healthcare11121783)
Supplement: Supplementary file 1 [file healthcare-11-01783-s001.zip › healthcare-2384044-supplementary.pdf]

Supplementary

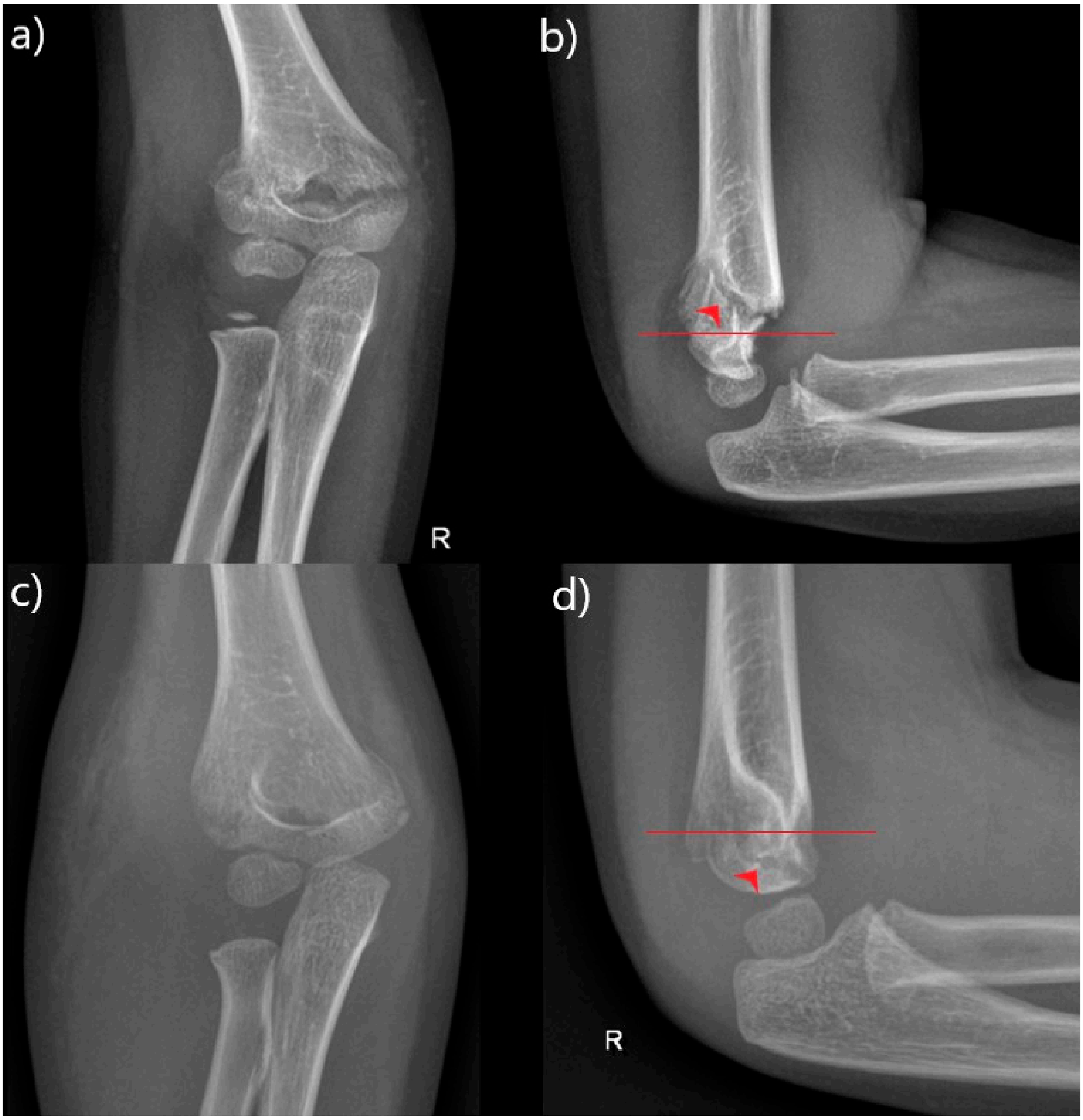

Figure S1. Anteroposterior and lateral x-rays of supracondylar fractures; (**a,b**) - high type of supracondylar fracture of the right humerus in a four-year-old boy; (**c,d**) - low type of supracondylar fracture of the right humerus in a five-year-old girl (red line - isthmus of the humerus, red arrow - fracture)
